# Influence of Surface Forces on Membrane Separations

**DOI:** 10.3390/membranes12040400

**Published:** 2022-04-02

**Authors:** Anatoly N. Filippov, Huseyin Selcuk

**Affiliations:** 1 Department of Higher Mathematics, Gubkin Russian State University of Oil and Gas, Leninsky Prospect 65-1, Moscow 119991, Russia; 2 Department of Environmental Engineering, Faculty of Engineering, Istanbul University-Cerrahpasa, Avcilar, Istanbul 34320, Turkey

This Special Issue of *Membranes*, entitled “Influence of Surface Forces on Membrane Separation”, is published in honour of Professor Victor Mikhailovich Starov, Doctor of Science in Chemistry, Fellow of the Royal Society of Chemistry. Victor is a Professor in the Department of Chemical Engineering, Loughborough University, UK. 14 June 2021 was the 75th birthday of Professor Starov (Figure 1).

Victor graduated from Moscow State University in 1969. He was awarded his PhD entitled “Capillary Hysteresis and the Structure of Isotropic Porous Media” in 1973 by the USSR Academy of Sciences and his Doctor of Sciences, “Equilibrium and Kinetics of Thin Liquid Layers”, awarded in 1981 by Petersburg University. Since 1974, Victor has collaborated in his research with the Fellow of the Russian Academy of Sciences, B.V. Derjaguin and Profs. N.V. Churaev, V.D. Sobolev, as well as other colleagues from the Institute of Physical Chemistry, USSR Academy of Sciences (now Frumkin Institute of Physical Chemistry and Electrochemistry, Russian Academy of Sciences).

In 1983, Victor became the head of the Department of Pure and Applied Mathematics, Moscow State University of Food Production, where, in addition to teaching, he became involved in collaborative research with his colleagues A.N. Filippov, V.V. Kalinin, V.I. Ivanov, S.I. Vasin, and Yu.E. Solomentsev. After the collapse of the Soviet Union, Victor worked for about three years as a Visiting Professor in the Department of Chemical Engineering, University of Texas, Austin (US), and then at Toulouse University (France), Instituto Pluridisciplinar (Madrid, Spain), and Swansea University (Wales, UK). In 1998, he permanently moved to the Department of Chemical Engineering, Loughborough University (UK) as a Professor in Chemical Engineering.

For many years, Victor was involved in various aspects of membrane separation, including reverse osmosis, ultra- and microfiltration, and electrodialysis. He organised Special Issues for the journals *Advances in Colloid and Interface Science* and *Journal of Membrane Science*, which published a collection of papers by membrane scientists from Russia and later co-organised the publication of a Special Issue of the *Desalination* journal with Professor Gangasalam Arthanareeswaran, which published a collection of papers presented by membrane scientists from India. In his innovative research of reverse osmosis, Victor determined the maximum value of the rejection coefficient and corresponding optimum velocity of filtration, which were calculated through all physicochemical parameters of the process. He explained the negative rejection of some ions from mixtures, as well as a change in the pH of the permeate. Victor determined that the streaming potential was calculated as a function of Peclet number, distribution coefficients, and membrane charge. A theory of reverse osmosis of multicomponent electrolyte solutions was developed and verified using the available experimental data, which demonstrated a very good agreement between the theory predictions and experimental data. Victor developed a theory of the formation and compressibility of cake layers formed on membrane surfaces during ultra- and microfiltration based on colloidal interactions between particles and membranes. He also suggested a new theory of the sieve mechanism of microfiltration, which was verified by experimental investigations. A new phenomenon of the concentration of electrolytes in the permeate during ultrafiltration in the presence of polyelectrolytes in the feed solution was predicted and verified. Victor investigated the electrodialysis of bilayer membranes, and his predictions were verified against experimental data.

Victor also studied in detail the simultaneous action of capillary forces and disjoint pressure in thin liquid layers and a transition zone (in the vicinity of liquid layers near the three-phase contact line) under equilibrium, quasi-equilibrium, and dynamic conditions. This made it possible to identify the stability conditions of the transition zone, in order to predict the existence and stability of equilibrium non-flat liquid layers. This made it possible to investigate the influence of solid surface roughness on the measured values of the disjoining pressure, and to predict an instability threshold for thin liquid layers on outer cylindrical or spherical surfaces in the case of complete wetting. He explained the phenomenon behind the static hysteresis of a contact angle on a smooth homogeneous surface, which allowed him to calculate both the advancing and receding contact angles via the disjoining pressure isotherm and to predict the presence of thick films behind a receding meniscus (the latter phenomenon was discovered and studied by N.V. Churaev and his group).

Victor expressed line tension via disjoining pressure and calculated its value, and he studied the deformation of soft solids caused by the disjoining pressure in the transition zone. He also predicted both the exponent and pre-exponential factors in the spreading law in the case of complete wetting (an excellent agreement between theory and experimental data was later disclosed). Furthermore, Victor investigated the motion of bubbles and oil droplets in thin cylindrical and/or tapered capillaries under the applied pressure or temperature gradient.

Together with his colleagues, Prof. Starov predicted the adsorption of surfactant molecules on bare hydrophobic surfaces in front of moving aqueous surfactant solutions. This allowed him to formulate and experimentally confirm the kinetics behind the spreading of surfactant solutions over hydrophobic surfaces and the imbibition of surfactant solutions into hydrophobic capillaries.

Victor developed a new theory of the spreading of liquids over dry and liquid-saturated porous substrates. Based on this theory, the universal law of spreading was predicted, which turned out to be in excellent agreement with experimental observations. He also developed a theory on foam drainage placed on a thin, porous substrate.

Victor developed a new theory on the effective viscosity of suspensions and emulsions for the consideration of cluster formation and proposed a new method of calculation of the effective properties of porous media.

Prof. Starov always invested a lot of effort into the training of young scientists. More than 30 PhDs and 4 Doctor of Sciences degrees were successfully awarded to his younger colleagues under his supervision in Russia, the United States, Bulgaria, Spain, France and the UK. V.M. Starov is a well-respected figure, both among his colleagues and all over the world. At present, he is the member of the editorial boards of 14 scientific journals, including *Desalination*, *Colloid*, *Advances in Colloid and Interface Science*, and *Current Opinion in Colloid and Interface Science*. He is an Editor-in-Chief of the *Journal of Chemical Engineering & Process Technology* and an Associate Editor of the *Journal of Colloids and Interfaces*. Prof. Starov is a Member of the Council of the International Association of Colloid and Interface Scientists, an Honorary Professor at Moscow State University of Food Production, and a Fellow of the Royal Society of Chemistry.

Prof. Starov has published around 300 papers: https://scholar.google.co.uk/citations?hl=en&user=HxpVycEAAAAJ&view_op=list_works (accessed on 31 March 2022). Please see the references for a collection of Victor’s most frequently cited works [1,2,3,4,5,6,7,8,9,10,11,12] related to spreading and wetting phenomena and membrane processes.

In this Special Issue, seven research articles are published, including one review paper [13] that emphasizes the main aspects of membrane distillation (MD) as a membrane-based, temperature-driven water reclamation process. This review assesses the advancements in the design of modified and novel MD configurations with an emphasis on the effects of upscaling and pilot-scale studies. The improved MD configurations discussed include the material gap MD, conductive gap MD, permeate gap MD, vacuum-enhanced AGMD/DCMD, submerged MD, flashed-feed MD, dead-end MD, and vacuum-enhanced multi-effect MD. All of these modified MD configurations are designed to either reduce heat loss by mitigating temperature polarization or to improve the mass transfer and permeate flux. It is highlighted that the comparison of various configurations is prevented by a lack of standardized testing conditions. The authors reflect on recent pilot-scale studies, the ongoing hurdles of commercialization, and niche applications of the MD process.

The aim of paper [14] is to establish and quantify the asymmetry of the current–voltage curve of a novel bilayer composite membrane based on a thick layer (219µm) of the cation-exchange perfluorinated membrane, MF-4SC (the Russian equivalent of Nafion^®^-117), and a thin (1µm) non-conducting layer of a glassy polymer of internal microporosity poly(1-trimethylsilyl-1-propyne) (PTMSP) depending on the direction of the external electric field. The authors have theoretically shown that bilayer membranes with the highest difference in the modules of effective exchange capacities have the greatest asymmetry. This means that one of the membrane layers must be neutral in order to reach a maximum asymmetry. Using the physicochemical characteristics of one-layer membranes, MF-4SC and PTMSP, the asymmetric current–voltage curves of the bilayer composites are described with a good accuracy up to the limiting regime start in 0.05 M HCl and NaCl solutions. This is because information about the electrochemical behaviour of such membranes in proton form is necessary to assess the prospects of their use as a proton-conducting membrane in a fuel cell. The layered membrane composites with a high asymmetry of current–voltage curves, such as MF-4SC/PTMSP, are important for potential applications in electromembrane devices, such as membrane sensors, detectors, and diodes.

The authors of [15] tested a novel photoelectrocatalytic membrane (PECM) reactor as an option for the desalination, disinfection, and detoxification of biologically treated textile wastewater (BTTWW), with the aim of reusing it in hydroponic farming. The anionic ion exchange (IEX) process was used before PECM treatment to remove toxic residual dyes. The toxicity evaluation for every effluent was carried out using the *Vibrio fischeri*, Microtox^®^ test protocol. The disinfection effect of the PECM reactor was studied against *E. coli*. After PECM treatment, the 78.7% toxicity level of the BTTWW was reduced to 14.6%. However, photocatalytic desalination during treatment was found to be slow (2.5 mg L^−1^ min^−1^ at 1 V potential). The reactor demonstrated approximately 52% COD and 63% TOC removal efficiency. The effects of wastewater reuse on hydroponic production were comparatively investigated by following the growth of the lettuce plant. A detrimental effect of reusing BTTWW was observed in the lettuce plant, while no negative impact was reported using the PECM-treated textile wastewater. In addition, all macro/micronutrient elements in the PECM-treated textile wastewater were recovered by hydroponic farming, and the PECM treatment could ay be an eco-safe wastewater reuse method for crop irrigation.

Yuzer and Selcuk [16] used the bipolar membrane electrodialysis (BPMED) process to recover wastewater and salt in biologically treated textile wastewater (BTTWW). The BPMED process, with and without pre-treatment (softening and ozonation), was evaluated under different operational conditions. Water quality parameters (colour, remaining total organic carbon, hardness, etc.) in the acid, base, and filtrated effluents of the BPMED process were evaluated for acid, base, and wastewater reuse purposes. Ozone oxidation decreased colour by 90% and chemical oxygen demand reduced by 37% in BTTWW. As a result, dye fouling on the anion exchange membrane of the BPMED process was reduced. Subsequently, over a 90% desalination efficiency was achieved in a shorter period. Generated acid, base, and effluent wastewater of the BPMED process were found to be reusable in the wet textile processes. The results indicated that pre-ozonation and subsequent BPMED membrane systems might be a promising solution for converging to a zero-discharge approach in the textile industry.

In [17], an approximate model based on friction–coefficient formalism was developed to predict the mixed-gas permeability and selectivity of polymeric membranes. More specifically, the model is a modification of Ora Kedem’s approach to flux coupling. The crucial assumption of the developed model is the division of the inverse local permeability of the mixture components into two terms: the inverse local permeability of the corresponding pure gas and the term proportional to the friction between penetrants. Analytical expressions for the permeability and selectivity of polymeric membranes in mixed-gas conditions were obtained within the model. The input parameters for the model are ideal selectivity and solubility coefficients for pure gases. Calculations have shown that, depending on the input parameters and the value of the membrane Peclét number (the measure of coupling), there can be both a reduction and an enhancement in selectivity compared to the ideal selectivity. The deviation between real and ideal selectivity increases at higher Peclét numbers; in the limit of large Peclét numbers, the mixed-gas selectivity tends to have the value of the ideal solubility selectivity. The model has been validated using literature data on the mixed-gas separation of *n*-butane/methane and propylene/propane through polymeric membranes.

The authors of [18] proposed a new method to increase rejection in microfiltration by applying membrane oscillation that uses a new type of microfiltration membrane with slotted pores. The oscillations applied to the membrane surface result in reduced membrane fouling and increased separation efficiency. An exact mathematical solution of the flow in the surrounding solution outside the oscillating membrane was developed. The oscillation resulted in the appearance of a lift velocity, which moved oil particles away from the membrane. The latter resulted in both reduced membrane fouling and increased oil droplet rejection. This developed model was supported by the experimental results of oil–water separation in the produced water treatment. It was proven that the oil droplet concentration was notably reduced in the permeate due to the membrane oscillation, and that the applied shear rate caused by membrane oscillation also reduced pore blockage. A four-times-lower oil concentration was recorded in the permeate when the membrane vibration frequency was 25 Hz, compared to without membrane vibration. Newly generated microfiltration membranes with slotted pores were used in the experiments.

In nanofiltration (NF) and reverse osmosis (RO) processes, aminophosphonates, such as aminotris(methylenephosphonic acid) ATMP prevented inorganic scaling leading to a more stable membrane performance. So far, as studied in [19], little attention has been paid to the possible permeation of aminophosphonates through NF and RO membranes. The authors investigated the permeability of these membrane types for ATMP and its potential metabolites iminodi(methylenephosphonic acid) (IDMP) and amino(methylenephosphonic acid) (AMPA) with two different NF membranes (TS40 and TS80), one RO membrane (ACM2) and three different water compositions (ultra-pure water, synthetic tap water and local tap water). They found traces of phosphonates in all of the investigated permeates. The highest phosphonate rejection occurred with local tap water for all three membranes investigated. Filtration experiments with a technical antiscalant formulation containing ATMP indicated similar trends of phosphonate permeability through all three membranes. The authors assume that the separation mechanisms of the membranes are the result of a very complex relationship between physicochemical properties, such as the Donnan exclusion, feed pH, feed ionic strength and feed concentration, as well as solute–solute interactions.

Thus, the articles in this Special Issue are devoted to innovative research of membranes and membrane processes, as well as their use in practical applications. The variety of research topics to which these articles are dedicated fully reflects the broad outlook of Professor Starov.

In conclusion, the editors would like to thank the authors and reviewers for their valuable contributions to this Special Issue and the editorial staff of *Membranes* for their help and support during the review process.

## Figures and Tables

**Figure 1:**
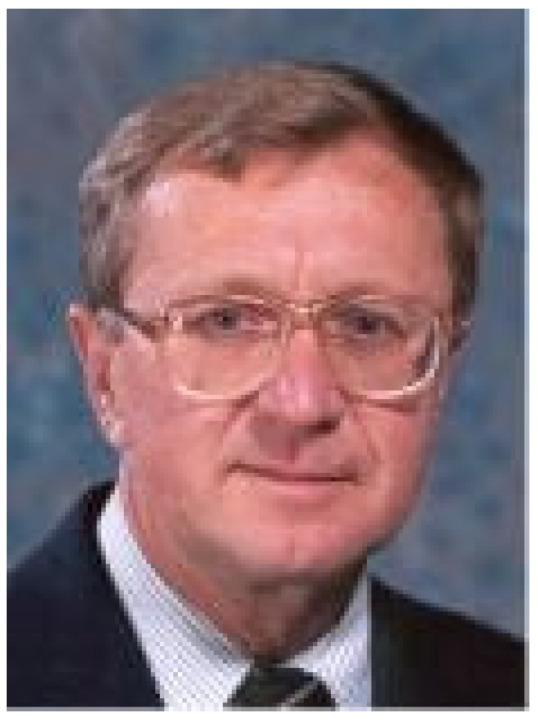
Professor Victor M. Starov.

## Data Availability

Not applicable.
